# Choline-Based Multi-Ingredient Supplementation Can Improve Explosive Strength during a Fatiguing Task

**DOI:** 10.3390/ijerph182111400

**Published:** 2021-10-29

**Authors:** Matthew Gage, Kevin Phillips, Byungjoo Noh, Tejin Yoon

**Affiliations:** 1 Department of Kinesiology and Integrative Physiology, Michigan Technological University, Houghton, MI 49931, USA; matthewg@gogebic.edu; 2 Career Program, Gogebic Community College, Houghton, MI 49931, USA; 3 Exercise Science Program, Brevard College, Brevard, NC 28712, USA; phillikc@brevard.edu; 4 Department of Kinesiology, Jeju University, Jeju-si 63243, Korea; bnoh@jejunu.ac.kr; 5 Department of Physical Education, Kangwon National University, Chuncheon 24341, Korea; 6 Interdisciplinary Program in Biohealth-Machinery Convergence Engineering, Kangwon National University, Chuncheon 24341, Korea

**Keywords:** supplement, fatigability, choline, rate of force, rate of torque, GPC

## Abstract

Various choline-based multi-ingredient supplementations (CMS) have been suggested in the current market, but the research is limited. The purpose of this study was to investigate the acute effect of a CMS on physical performance. Fourteen male college football players (20.4 ± 1.0 years) participated in a randomized double-blind crossover experiment separated by 7 days. Subjects were given a CMS or a placebo 60 min before physical performance testing measures, including maximum vertical jumps, maximum voluntary isometric contractions (MVIC), maximal voluntary concentric contractions (MVCC), and fatiguing contractions. Four MVICs and seven sets of two MVCCs at various loads (1 N·m to 60% MVIC torque) were performed with the knee extensor muscles while seated on a dynamometer before and after the fatiguing tasks. During the fatiguing tasks, 120 MVCCs (4 sets × 30 reps) were performed with a load equivalent to 20% MVIC. Twitch interpolation technique was used to assess muscle contractile properties and voluntary activation. No significant differences were seen at baseline between sessions for all testing measures including vertical jump height, strength, power, muscle contractile properties and voluntary activation. Rate of torque development and impulse was higher in supplemental session compared to control session throughout the fatiguing contractions (*p* = 0.018, *p* < 0.001, respectively). Acute CMS can improve explosive strength by delaying the onset of fatigue.

## 1. Introduction

In most sports, athletes are required to produce not only a great amount of force but also produce that force at a high rate in order to perform a variety of skills including sprinting, jumping, and changing of direction. Many athletes consider supplementation as a means of gaining a physical edge on their competition [1] with the highly competitive culture in today’s athletics. The prevalence of use of sports supplements has rapidly increased over the last decade. For example, the prevalence rate was 82.2% of young athletes in young athletes from 4 countries in 18 sports at the international level [2].

Among various supplements, a growing number of athletes seem to be supplementing with different types of Alpha-glycerophosphocholine (Alpha-GPC or A-GPC) supplement [3]. A-GPC is a putative acetylcholine (ACh) precursor [4] i.e., once ingested, it converts to phosphorycholine and then could serve as a source of choline for ACh synthesis. ACh plays an important role as a neurotransmitter in central, peripheral, autonomic, and enteric nervous system [5]. Particularly in the neuromuscular junction, Ach is released by an action potential from a motor neuron, causing the ion channels of muscle fiber to open, which is one of the important sequential steps to finally contract muscle. Based on this premise, various A-GPC supplementations have been suggested in the current market, but the experimental evidence is lacking. A few studies have recently investigated the effect of A-GPC on human cognitive and physical performance, but the results were not consistent. For example, significant improvement was found in bench press capability [6], but no acute effects (within ~60 min) were found in strength or power at baseline [7,8]. Bellar, LeBlanc and Campbell [7] found ~3% greater strength of thigh after 6 days of 600 mg A-GPC dose compared to the baseline, but the improvement was not significant in upper limb exercise. More recently, the same group performed a similar study with smaller doses (250 and 500 mg A-GPC) for seven days and found a significant improvement in maximal power performance during countermovement jump for 250 mg dose groups only [9]. The findings of these studies suggest that the effect of A-GPC on muscular performance is limited and might be dependent the amount and duration of dose.

In addition to the above studies, which tested with A-GPC only, a study demonstrated a possible positive effect of A-GPC by dosing with an additional ingredient, caffeine [10]. A recent study also found that multi-ingredient supplementation showed several positive effects during bench press exercise, subjective fatigue, and power [11]. These two studies suggest a possible benefit of choline supplementation with additional ingredients for better outcomes. For similar reasons, a CMS (Nutromic Sport Nutrition, Muskegon, MI, USA) contained mainly A-GPC along with a propriety blend of ingredients, including huperzia serrata, panax quinquefolius extract (American ginseng), and Bioperine^®^ black pepper extract. Particularly, Huperzine A, extracted from huperzia serrata is a well-known alkaloid that is an effective and highly specific inhibitor of acetylocholinesterase [12]. Huperzine A inhibits the acetylcholinesterase enzyme from breaking down acetylcholine, thereby increasing both the level and duration of action of the neurotransmitter acetylcholine [13]. The scientific evidence showing CMS has a positive effect on physical performance, however, is still lacking.

Although it appears difficult to draw firm conclusions, the effect of neurotransmitter on force generations during the fatiguing contractions [14], the decrease in quantity of Ach could be a limiting factor. Thus, it might be beneficial to dose CMS for those who need to repeat the high and fast contractions during which the level of choline could be declined quickly. The purpose of this study was to examine the effects of a choline-based multi-ingredient supplementation on physical performance in collegiate football players using both practical and laboratory-based testing measures at baseline and during fatigue contractions. We hypothesized that subjects would show an increase in physical performance as measured by vertical jump height, strength, rate of torque development (RTD), impulse, and power with CMS during a fatiguing task but not at baseline [7,8].

## 2. Materials and Methods

### 2.1. Experimental Approach to the Problem

**Figure 1:**
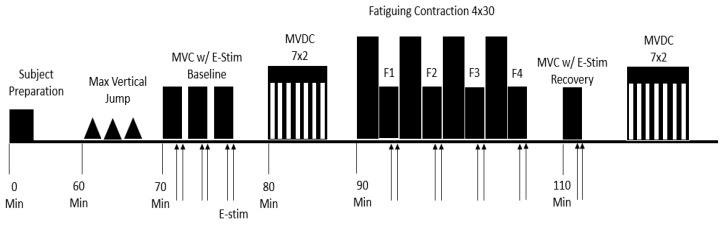
Experimental protocol for both control and supplemental sessions. Upward arrows denote electrical stimulation during maximum voluntary isometric contractions (MVIC) and rest. Hatched bars denote maximal voluntary concentric contractions (30 reps) with a load equivalent to 20% MVIC.

The experiment took place over a 10-week period while the athletes were participating in their summer strength and conditioning program, from June to July. During this time of the year, the athletes are not taking part in competition, and the primary focus is on physical performance training. This study was designed as a randomized double blind crossover trial in which each participant received two capsules of CMS or placebo and then crossed over to the alternate regimen. The testing consisted of two sessions: 1) the control session where subjects were given a placebo; 2) an experimental session where subjects were given two capsules of CMS. Sessions were separated by at least seven days to allow for a wash-out period and most of participants (~80%) were tested at the same time of the day. Participants were asked to refrain from any strenuous activity outside of their controlled team workouts for 24 h before each session. All sessions were completed in the human biomechanics lab on site near their training facility where the temperature was set ~25 to 27 °C during the experiments. All participants were warmed up by cycling with no load for 5-min before testing. The testing protocol consisted of several baseline measures including: 3 maximal vertical jumps to determine lower body power; 3 maximal voluntary isometric contractions (MVICs) to determine maximum isometric strength of the knee extensors; 7 sets of 2 repetitions of maximal voluntary concentric contractions (MVCCs) to determine lower body power. Fatiguing contractions were then completed followed by reassessment of baseline measures 10 min post completion of fatiguing task. See Figure 1.

### 2.2. Participants

Fourteen male Division 2 college football players (age 20.4 ± 1.0 years) volunteered to participate in the study. The number of participants was determined using G*power software for the statistical power 0.8 [15]. Participants were free from musculoskeletal disorders that would impair their ability to exercise. All participants completed a physical activity questionnaire [16] and were screened to have been highly active. A handedness questionnaire [17] was also administered to determine dominance. All participants had completed at least one academic school year and had been training regularly with the team’s strength and conditioning staff. All participants were able to understand experimental procedures and they were familiarized with main testing measures including dynamometer and electrical stimulations. A report of the participant’s characteristics can be found in Table 1. This study was approved by the Michigan Technological University’s Institutional Review Board for the Protection of Human participants. Informed consent was obtained by each individual before their participation commenced.

### 2.3. Procedures

*CMS.* All supplementation occurred in the presence of the researchers immediately when participants reported to the testing session. participants were given two capsules of CMS (1076 mg/capsule) or two capsules of fiber as a control in random order 60 min before beginning any of the physical testing protocol. Participants were strongly asked not to consume any caffeine or pre-workout supplements and keep their normal dietary on the day of their testing session.

*Maximum Vertical Jump.* Vertical jump was assessed using a Vertec (Vertec, JumpUSA, Sunnyvale, CA, USA) for a practical field measurement. First, the participant was asked to walk under the Vertec with both arms fully extended overhead in order to obtain the participants standing reach, participants then performed 3 maximum vertical jumps separated by one minute each.

*Experimental Setup and Data Recording.* Experimental sessions were performed with the participant seated on a Biodex multi-joint dynamometer (System 4 Pro; Biodex Medical System, Shirley, NY, USA). Biodex isokinetic dynamometer is mechanically reliable for the valid measurement of isometric torque and angular velocities (ICCs > 0.99) [18]. The reliability of our system was also verified by coefficient of variation for torque and power measurements (<2.5%). Each participant was seated in a slightly reclined position with the hip and knee angle at 95° and 90°, respectively. Participant’s shank was strapped to the distal end of the Biodex lever, with the lateral epicondyle of the femur aligned with the axis of rotation of the dynamometer. All voluntary and evoked isometric contractions were performed at 90° of flexion (0° being where the knee joint is fully extended and the lower limb horizontal). This angle was kept consistent in order to compare relative changes in angle and force production throughout the testing sessions [19]. Shortening (concentric) contractions moved through a 60° range of motion from 90° to 30° of flexion. Torque, angle, and angular velocity data were sampled at a rate of 2000 Hz using a micro 1401 AD converter and Spike 2 software [Version 8, Cambridge Electronics Design (CED), Cambridge, UK]. Torque signal was displayed on a 70-in TV monitor (Sharp Electronics, NJ) located 2.5 m in front of the participant.

*Electromyography and Electrical Stimulation.* Surface electromyography (EMG) system (Bagnoli 16; Delsys, Natick, MA, USA) was used to record activity of the knee extensor muscles, including the rectus femoris, vastus lateralis and vastus medialis throughout the testing. Electrode placement was determined according to recommendations by the Surface Electromyography for the Non-Invasive Assessment of Muscles (SENIAM Project) [20]. The ground electrode was positioned over the patella. The skin was thoroughly scrubbed with alcohol-soaked cleansing cloths before electrode placement, and location was marked via a permanent pen to ensure placement was consistent for the entirety of the testing sessions. The EMG signal was sampled at a rate of 2000 Hz using a micro 1401 AD converter and Spike 2 software. Electrical pulse (singlet, 100-μs duration) was applied to elicit twitch response of knee extensor muscles using a computer-controlled stimulator (D185; Digitimer, Welwyn Garden City, UK) and a pair of self-adhesive surface electrodes (6.98 × 12.7 cm, Dura-Stick plus DJO Brands). A more detailed description of electrode positions and stimulation protocol can be found in our previous paper [21].

*Maximal Voluntary Isometric Contractions*. Four MVICs were performed, each with a contraction time of 3–5 s using a Biodex dynamometer. Two minutes were given as a rest period in between contractions to ensure adequate recovery. Visual feedback of the live torque-time tracing was displayed for the participants on a 70-inch TV monitor, as well as verbal encouragement to ensure maximal effort during all MVICs. Electrical stimulation was applied to elicit twitch torque at the peak torque level during MVIC. An additional twitch was triggered upon relaxation (approximately 1 s) after the MVIC.

*Maximal Voluntary Concentric Contractions*. To measure muscle power, seven sets of isotonic contraction at various loads were performed, including 1 N·m, 10%, 20%, 30%, 40%, 50%, and 60% of MVIC. These loads were given in a randomized order, and stayed in the same randomized order for the duration of the testing procedures per individual. Participants were instructed to move the resistance load as “fast and hard” as possible throughout the full 60° range of motion (30–90°). Participants were provided with verbal encouragement and real-time torque feedback displayed on a TV monitor to encourage a maximal effort. Two consecutive repetitions at each resistance were performed to improve the chances that true maximal velocity was reached. Thirty seconds of rest were allotted in-between sets.

*Fatiguing Contractions.* Participants performed 4 sets of 30 repetitions of a dynamic leg extension at a constant load. The load used for these contractions was set to 20% of the Participants’ MVIC torque value. Similar to the MVCCs, participants were instructed to extend their leg as fast and as hard as possible throughout the entire 60° range of motion. After each extension, the dynamometer returned the participants’ leg passively to perform the next contraction. In between each set of 30 repetitions, participants performed one MVIC with electrical stimulation in order to assess the participant’s voluntary activation (VA) level.

*Recovery Measurements*. One MVIC and 7 sets of 2 MVCCs were performed 10 min after completing the fatiguing contractions.

To minimize potential sources of bias, all devices were calibrated properly and the functionality were verified by research assistants before the experiment. During the experiment, our well-trained testers kept monitoring participant’s facial expression and other behavioral responses, particularly during electrical stimulation.

### 2.4. Data Analysis

Data analysis of torque, angular velocity, power and EMG signals was performed offline with Spike 2 software. The torque signal was low-pass filtered with a 10 Hz cutoff frequency using embedded filter in Spike 2 program. Maximal strength was quantified as the mean torque of 100-ms duration before electrical stimulation during the MVIC. Rate of torque development was calculated as the peak tangential torque using a moving mean method of the torque-time curve over the first 400 ms from the onset of contraction [22]. Impulse was calculated using the area under curve of torque signal during 0–25, 25–50, 50–75, 75–100, 100–150 and 150–200 ms [23]. The validity and reliability of the Biodex dynamometer used to measure the torque have been reported in the literature [18].

Resting twitch (RT) produced by electrical stimulation in a relaxed but potentiated muscle (one second after the MVIC) was used to calculate the peak amplitude of twitch torque, time to peak torque (TTP), and half relaxation time (HRT). Both VA and corrected VA were calculated using the peak amplitude of the interpolated twitch (IT) and the RT with equations used in previous studies [24,25].

Knee extension power was calculated across each of the seven resistance loads, with the peak power being the highest product of torque and angular velocity at any given time-point during each MVCC. Knee extensor power during the fatiguing contractions was calculated by averaging the first and last three MVCCs.

EMG activity of the knee extensor muscles was determined as the root mean squared (RMS) value over a 100-ms interval, which was the time interval used to determine the MVIC torque. EMG was calculated as the RMS values of the corresponding time interval used to calculate impulse. All subsequent RMS EMG values were normalized to the level obtained during the baseline MVIC.

### 2.5. Statistical Analysis

Normality and homogeneity were tested using Shapiro-Wilk and Levene tests, respectively. All variables were normally distributed and the variances of variables for two sessions were equal (all *p*s > 0.05). A paired *t*-test was used to compare vertical jump height between control and supplemental sessions. A two-way Analysis of Variance (ANOVA) with repeated measures was used to compare dependent variables between sessions (control, supplement) and across time points (Baseline, MVIC, F1, F2, F3 and F4 for fatigue; F4 and Recovery to measure recovery respectively). The variables include MVIC, RTD, impulse, power, EMG, and VA. For each ANOVA the sphericity of data was verified with Mauchly’s test, and technical corrections were performed whenever necessary. To examine the qualitative meaning of the observed changes in the main variables, Cohen’s d effect size was calculated (>0.2, small; >0.5, moderate; >0.8, large). Statistical Package for the Social Sciences (SPSS) software version 22 (IBM, Armonk, NY) was used for all statistical analysis. A significance level of *α <* 0.05 was used to identify statistical significance.

## 3. Results

### 3.1. Vertical Jump

Maximum vertical jump showed no significant difference in jump height between control and supplemental sessions (70.8 ± 6.6 vs. 70.9 ± 6.2 cm, *t*_13_ = 0.135, *p* = 0.895).

### 3.2. MVIC Torque

Maximal voluntary isometric contraction torque at baseline was similar between control and supplemental sessions (297.8 ± 48.4 vs. 296.7 ± 70.5 N·m; *t*_13_ = 0.088, *p* = 0.931). Throughout the fatigue and recovery, MVIC changed similarly between two sessions (*p* = 0.839). EMG of VL and VM changed throughout the session similarly between two sessions (*p* > 0.05). EMG activity of the RF during 0-25 ms of MVIC contractions after the first 30 fatiguing contractions was significantly greater in supplementary session than control session (8.4 ± 3.5 vs. 10.4 ± 3.1% of MVIC, *t*_13_ = 1.135, *p* = 0.029, Cohen’s *d* = 0.605).

### 3.3. Rate of Torque Development and Impulse

**Figure 2:**
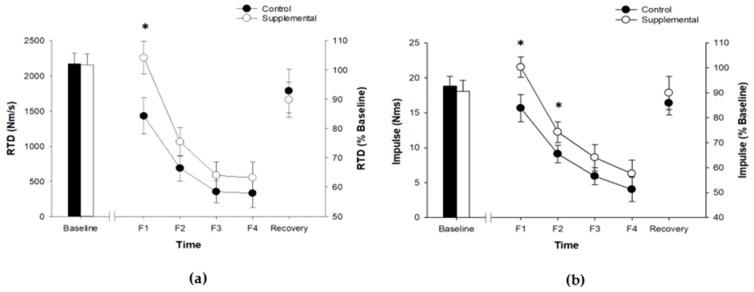
Rate of torque development during first 400 ms of each MVIC (**a**); Impulse during the first 200 ms of each MVIC (**b**). F1–F4 denotes ~10 s time duration when MVIC was performed during and after fatiguing tasks. Values are mean ± SE, presented with both absolute (left axis) and relative (right axis) scales. *—Significant difference between two sessions (*p* < 0.05).

Rate of torque development at baseline was similar between control and supplemental sessions (2171 ± 564 vs. 2156 ± 567 N·m/s; *t*_13_ = 0.140, *p* = 0.891). RTD decreased during the fatiguing contractions (fatigue effect; *F*_2.0_, _26.6_ = 27.0, *p* < 0.001, *η*^2^*p* = 0.675), and the decline was similar throughout the testing protocol for both sessions (fatigue × session; *F*_4, 52_ = 2.23, *p* = 0.079, *η*^2^*p* = 0.146). RTD was significantly higher in the supplemental session compared to control session (session effect; *F*_1.0, 13.0_ = 7.40, *p* = 0.018, *η*^2^*p* = 0.363), but it was significant at the end of the first set (F1) only (1849 ± 480 vs. 2244 ± 453 N·m/s; *t*_13_ = -2.914, *p* = 0.012, Cohen’s *d* = 0.605). After 10 min of recovery, RTD significantly increased (recovery effect; *F*_1, 13_ = 54.37, *p* < 0.001, *η*^2^*p* = 0.807), and the relative increase was similar between sessions (recovery × session; *F*_1, 13_ = 1.96, *p* = 0.216, *η*^2^*p* = 0.115). See Figure 2a.

All impulse values at baseline were similar between control and supplemental sessions (*p* > 0.05). All impulse values decreased throughout the fatiguing contractions (fatigue effect; *p* < 0.001), and the decline was similar throughout the testing protocol for both sessions (fatigue × session; *p* > 0.05). Despite the similar decrease for impulse throughout the fatiguing protocol, impulse was greater for participants during the supplemental session when compared to the control session throughout the fatiguing contractions (session effect; *p* < 0.001, *η*^2^*p* = 0.457), but it was significant at the end of the first and 2nd sets (F1 and F2 only; *p* = 0.006, Cohen’s *d* = 0.901; *p* = 0.032, Cohen’s *d* = 0.351, respectively). After 10 min of recovery, impulse increased significantly (recovery effect; *p* < 0.001), and the relative increase was similar between sessions (recovery × session; *p* > 0.05). The changes in impulse at different time intervals were similar. The impulse during the 0–200 ms was drawn as representative data. See Figure 2b.

### 3.4. Muscle Contractile Properties and Voluntary Activation

Throughout the fatigue and recovery VA, and contractile properties changes similarly between two sessions (*p* > 0.05). See Table 2.

### 3.5. Power

Knee extensor power showed no significant differences across all loads between control and supplemental sessions (*F*_1,13_ = 3.77, *p* = 0.074, *η*^2^*p* = 0.225). Mean power through the fatiguing contractions decreased similarly between control and supplemental sessions (*F*_1,13_ = 1.37, *p* = 0.263, *η*^2^*p* = 0.095).

## 4. Discussion

This study investigated the effect of choline-based multi-ingredient supplement on physical performance in division two college football players. The main findings of this study were that 1) acute supplementation showed no improvements in MVIC strength, RTD, power, and jump height at baseline; 2) acute supplementation delayed the onset of fatigue for RTD and impulse; 3) The decreased fatigability of static explosive force production (RTD and impulse) could be explained by the increased muscle activity.

A limited number of studies have examined the acute effect (30 to 90 min) of A-GPC on physical performance at baseline, but the results have varied. For instance, a study observed ~14% increase of bench press force [6], but no other studies found differences in vertical jump performance [8], or isometric strength [8] or peak power during a vertical jump [26] after the acute supplementation of A-GPC. Similar to the previous studies, the current study did not find any improvements in physical performance measured by MVIC, power, RTD, impulse, and vertical jump height at baseline. In the current study, although no dietary food log was recorded, participants were strongly asked to keep their normal dietary on the day of testing sessions so that the level of choline could be similar between two sessions. In the absence of disease, choline concentration does not limit the synthesis of ACh. Therefore, it is reasonable to assume that our participants already had adequate levels of ACh at baseline and the supplementation to increase the amount of ACh served no effect on initial performance. Another possible explanation for not finding significant improvements could be the high level of training of our participants. For example, a recent study tested the normal healthy college-aged males after six days of 600 mg A-GPC supplementation and found a significant increase in lower body isometric strength [7], the improvement was just ~3%. Our participants were division 2 football players and relatively well trained. In line with this, our participants were able to fully activate their knee extensor muscle fibers during MVIC (corrected VA > 98%). The previous findings and our results suggest that the acute effect of A-GPC on muscular functions at baseline when the normal choline level is maintained could be limited or negligible.

Oral choline supplementation might only increase endurance performance in activities that reduce circulating choline levels below normal [27]. Thus, many studies examined the effect of choline ingredient supplementation on fatigue [28,29,30]. For example, Spector and colleagues tested 20 trained cyclists during brief and prolonged cycling exercise with and without a choline supplemented drink. Fatigue times and work performed under either conditions were similar [29]. Another study discovered that soldiers did not have any benefit from choline supplementation when carrying heavy loads over long distances (~4 hrs) on a treadmill [30]. Serum or plasma choline levels were measured in these studies, and both studies found choline was not depleted because participants were highly trained cyclists [29] or although plasma choline increased with the supplement it was also maintained under the placebo condition [30]. Regardless of the duration of exercise, the cycling and walking exercise protocol might not deplete choline levels in these studies. Thus, choline supplement did not improve endurance in highly trained cyclists (VO_2max_: 58 to 81) and soldiers. Compared to these studies, our protocol (Total 120 maximal contractions during the 4 min period) was shorter but much more intense because maximal strength decreased ~40–50% after the fatiguing contractions. Although we did not measure the choline level directly, it might have become depleted, therefore affecting the release of ACh during muscular contractions [31]. Due to the increased intensity of the current protocol, some positive effects of supplementation were expected. We found that RTD and impulse during isometric MVICs were significantly greater during the fatiguing protocol of the supplementation session. However, RTD at one time point (F1) and impulse at two time points (F1 and F2) of 4 time points were significantly greater during the fatigue protocol. In addition, no differences in RTD and impulse were found at 10 min recovery. These results suggest that the effect were limited on the early stage of fatigue. We also expected that maximal strength would improve similarly with RTD, because improvements in RTD are led by increases in maximal strength [22]. However, MVC torque was similar during and after fatiguing tasks between sessions, which is consistent with a previous study [28]. Inconsistent results between MVIC and RTD could be explained by the fact that MVIC and RTD potentially are governed by different physiological mechanisms [32]. Similar to MVIC, mean power during fatiguing contractions were not different between control and supplement sessions. Unfortunately, we are not able to explain why no differences were found in power, we just postulate power measurement was not sensitive enough to find the difference.

It is known that both neural and muscular factors contribute to RFD [32]. In the current study, muscle contractile properties measured by twitch amplitude, TTP and HRT were similar between two sessions, and other muscular factors such as muscle size, architecture, and fiber types are not likely to change with acute supplementation. Although there could be substantial inter-individual variability in force production [30], the higher RTD and impulse (~10% respectively) found in the current study could be explained by improved functions at the neuromuscular junction due to the increased availability of acetylcholine due to supplementation.

Although no differences were found in voluntary activation throughout the current protocol, we did find greater EMG activity for rectus femoris muscle during a very early phase of contraction (0–25 ms). This result is interesting, because it showed that EMG activity increased without the increase of voluntary activation, suggesting that measurements of voluntary activation is not sensitive enough to find the difference in EMG, particularly during short time periods (<200 ms). Generally, increases in RTD are associated with the increased muscle activation [19,33]. However, it is still not clear how our participants increased muscle activation more during the supplemental session than the control session in the current study, because the increased EMG was not correlated with the increased impulse in the current study. The improved communication between the motor nerves and muscle fibers at the neuromuscular junction could be a possible explanation for the increased EMG activity thus improved RTD and impulse.

A few limitations of this study should be noted. First, because no dietary food log was recorded, it is not sure that the level of choline at the baseline are similar between two sessions. Second, we were not able to compare the effect between A-GPC only and A-GPC with other ingredients. Finally, we were not able to analyze the m-wave, an index of integrity of neuromuscular junction [34], due to technical issues. Thus, a future study should include recording dietary food log, and additional session with A-GPC only. In addition, nerve stimulation should be used to have m-wave data.

## 5. Conclusions

This study is the first study to investigate the effect of A-GPC supplementation with the additional ingredients (inhibitor of acetylocholinesterase) on physical performance and the underlying mechanism. Although we observed the significant increase in explosive strength and impulse during the maximal intensity fatiguing contractions, it was significant only at the early stage. In addition, no significant improvements were found at various baseline measurements.

Based on the results from the current study, which showed an increased RTD and impulse throughout a fatiguing task, athletes could potentially improve static explosive strength in their sport and training by supplementing with CM.

## Figures and Tables

**Table 1:** Subject Characteristics.

Variables (n = 14)	Values
Age (years)	20.4 ± 1.04
Height (cm)	191.4 ± 5.5
Weight (kg)	106.9 ± 16.4
Body fat (%)	17.7 ± 5.4
Physical activity (MET h/wk)	139.2 ± 55.9
Handedness (a.u)	40.7 ± 65.4
BMI (kg/m^2^)	29.1 ± 3.8

Note: MET-metabolic equivalent of task. Values are presented as mean ± SD.

**Table 2:** Muscle contractile properties and voluntary activation.

Variables	Control Session (n = 14)			Supplemental Session (n = 14)			Session Effect *p*-Value, Effect Size (*η*^2^*p)*
	Baseline	Fatigue	Recovery	Baseline	Fatigue	Recovery	
Time to peak (ms)	108.7 ± 7.2	122.0 ± 12.0	116.9 ± 28.2	109.4 ± 7.9	123.6 ± 9.3	113.3 ± 7.6	0.09, 0.20
HRT (ms)	49.2 ± 5.5	67.2 ± 31.0	48.2 ± 14.7	48.2 ± 7.0	77.7 ± 31.8	50.3 ± 10.9	0.33, 0.08
RT (Nm)	75.0 ± 13.6	27.1 ± 9.5	52.8 ± 15.8	75.6 ± 15.4	29.3 ± 11.9	52.3 ± 14.8	0.22, 0.11
VA (%)	99.2 ± 1.09	98.7 ± 1.75	97.3 ± 3.40	98.1 ± 1.72	97.4 ± 3.74	97.8 ± 2.53	0.18, 0.15

Note: VA-voluntary activation; RT-resting twitch; HRT-half relaxation time. Values are presented as mean ± SD.

## Data Availability

The data presented in this study are available in this manuscript.
